# Best Practices to Maximize the Use and Reuse of Quantitative and Systems Pharmacology Models: Recommendations From the United Kingdom Quantitative and Systems Pharmacology Network

**DOI:** 10.1002/psp4.12381

**Published:** 2019-03-22

**Authors:** Lourdes Cucurull‐Sanchez, Michael J. Chappell, Vijayalakshmi Chelliah, S. Y. Amy Cheung, Gianne Derks, Mark Penney, Alex Phipps, Rahuman S. Malik‐Sheriff, Jon Timmis, Marcus J. Tindall, Piet H. van der Graaf, Paolo Vicini, James W. T. Yates

**Affiliations:** ^1^ GlaxoSmithKline Medicines Research Centre Stevenage UK; ^2^ School of Engineering University of Warwick Coventry UK; ^3^ Certara QSP Canterbury UK; ^4^ Quantitative Clinical Pharmacology Early Clinical Development Innovative Medicines and Early Development Biotech Unit AstraZeneca Cambridge UK; ^5^ Certara Princeton New Jersey USA; ^6^ Department of Mathematics University of Surrey Guildford UK; ^7^ Union Chimique Belge‐Celltech Slough Berkshire UK; ^8^ Pharmaceutical Sciences Roche Pharmaceutical Research & Early Development Roche Innovation Center Welwyn Garden City UK; ^9^ European Molecular Biology Laboratory European Bioinformatics Institute Wellcome Genome Campus Cambridge UK; ^10^ Department of Electronic Engineering University of York York UK; ^11^ Department of Mathematics and Statistics University of Reading Reading UK; ^12^ The Institute for Cardiovascular and Metabolic Research University of Reading Reading UK; ^13^ Leiden Academic Centre for Drug Research Leiden The Netherlands; ^14^ Clinical Pharmacology Pharmacometrics and Drug Metabolism and Pharmaco‐Kinetics MedImmune Cambridge UK; ^15^ Development Sciences Kymab Ltd Cambridge UK; ^16^ Drug Metabolism and Pharmaco‐Kinetics, Oncology, Innovative Medicines and Early Development AstraZeneca Chesterford Research Park Cambridge UK

## Abstract

The lack of standardization in the way that quantitative and systems pharmacology (QSP)  models are developed, tested, and documented hinders their reproducibility, reusability, and expansion or reduction to alternative contexts. This in turn undermines the potential impact of QSP in academic, industrial, and regulatory frameworks. This article presents a minimum set of recommendations from the UK Quantitative and Systems Pharmacology Network (UK QSP Network) to guide QSP practitioners seeking to maximize their impact, and stakeholders considering the use of QSP models in their environment.

One of the key advantages of quantitative and systems pharmacology (QSP) modeling is its integrative and modular nature. This modular nature renders QSP an optimal scenario to reuse models by expanding them through the addition of more submodules, by reducing the number of submodels, or by translating existing modules to different contexts.

QSP is gaining traction against the background of the exponential increase in the number of QSP publications in scientific journals since the release of two seminal papers^1^, ^2^ (see **Figure** 1), the emergence of the first scholarly journal dedicated to systems pharmacology,^3^ and the growing number of mathematical models of biology readily available through public databases (BioModels,^4^ CellML,^5^ DDMore^6^). However, QSP is only slowly climbing up the innovation trigger slope of the Gartner Hype Cycle,^7^ which describes the maturity, adoption, and social application of emerging technologies through five consecutive phases (innovation trigger, peak of inflated expectations, trough of disillusionment, (positive) slope of enlightenment, and plateau of productivity). The consistent integration of QSP into the Model‐Informed Drug Discovery and Development (MID3) strategy within the pharmaceutical industry still proves challenging. In addition, the growth rate of the number of QSP models in public databases is slow compared with the increase of QSP publications, as a result of the lack of resources, inadequate model documentation, and a decline in the direct submission of models to those repositories by their authors. Published models tend to be generated *de novo* rather than being the result of an expansion or modification of existing models. This situation is, to a significant extent, because of the lack of standardization in the way that QSP models are developed, tested, and documented when made available to the public community, which hinders their reproducibility, reusability, and expansion or reduction to alternative contexts. This gap has been somewhat quantified by Kirouac *et al*.,^8^ who found that only 4 out of 12 model code files published in *CPT: Pharmacometrics & Systems Pharmacology* until October 2018 was executable. Transparency in reporting each stage of the modeling process and the adoption of good practice in doing so would greatly increase the likelihood of a model being more widely adopted and used by others.^9^ This is particularly important if QSP is to become a standard accepted approach as part of regulatory submissions, as from a regulatory perspective, transparency and collaboration increase the validation and acceptance of a model. Regulatory reviewers of QSP models face the challenge of having only 1 to 3 months to reproduce the results, evaluate underlying assumptions, and test the model with other/new data.^10^

**Figure 1:**
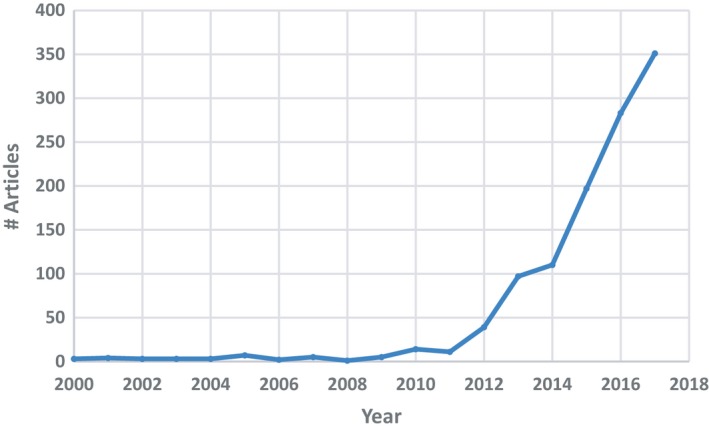
Annual number of PubMed abstracts containing the term “systems pharmacology” since the year 2000.

The ideal scenario is well illustrated by the filing in 2013 of a recombinant human parathyroid hormone (rhPTH) for the treatment of hypoparathyroidism.^11^ The US Food and Drug Administration (FDA) reviewers raised some concerns about the hypercalciuria being observed in the clinical study and used a publicly available calcium homeostasis QSP model^12^ to explore alternative dosage regimens. This QSP model was built on two earlier published models: a model of systemic calcium homeostasis^13^ and a cellular model of the behavior of bone morphogenic units.^14^ The QSP simulations supported the hypothesis that increased dosing frequency or slow infusion could reduce hypercalciuria. This led the FDA to request from the sponsors a postmarketing clinical trial to evaluate the effects of dose and dosing regimens on the control of hypercalciuria, and to use a mechanistic model–based assessment of prior pharmacokinetic/pharmacodynamic data to design this clinical trial.^15^ Had it not been for the publication and adequate development and description of those three models, the FDA would have missed the insight that led them to this impactful decision.

There are several common issues encountered by the modeling community when accessing models either from databases or peer‐reviewed publications, which stem from this lack of documentation standards. They can be summarized as follows:

1. *The purpose and/or scope of the model is unclear and/or the underlying assumptions are either poorly or not stated*. Often the structure of the model is provided without referring to the specific question or questions for which that model is intended. In other instances, the assumptions made to derive that structure or some of the parametric relationships are undeclared or vaguely described. The negative impact of poor descriptions of assumptions increases when the number of assumptions is high, which can be daunting to decision makers and collaborators. These situations make it difficult for the reader to assess the contexts for which the model would be suitable for and/or to what extent it can be adapted to similar contexts.
2. *The model and/or the publication lacks quantitative information*. Some models published in the mathematical biology space consist of little more than schema or networks describing the interactions between different players in biological or pharmacological systems, without any numerical information to inform on the frequency, extent, or rate of those interactions. Even in cases when the model is more quantitative, its parameter values, including units and uncertainty estimates, are often omitted from the publication or scattered inconsistently over text and figures. When those parameters are reported, the data, knowledge, and assumptions that underwrite their values are often missing or their adequacy, quality, and quantity are poorly discussed.
3. *The paper in which the model is published provides its description but fails to provide the associated model file or programming code*. The emergence of standardized markup languages (SBML),^16^ such as systems biology markup language,^9^ cell markup language (CellML),^5^ pharmacometrics markup language PharmML,^17^ or model description language (MDL),^18^ the existence of fully compatible modeling software (e.g., COPASI,^19^ SimBiology^20^), and the availability of open source tools to translate between them (e.g., Systems Biology Workbench^21^) should enable and encourage researchers to share their encoded model. However, although the provision of model files or computer code is encouraged by several scholarly journals, in practice some scientists are reluctant to give up their competitive advantage over other research groups. Some authors provide a high‐level description of the model to communicate their findings without necessarily including sufficient information to allow others to reproduce their findings.
4. *The model file or code is provided, but it is not properly documented*. Sometimes the code is inadequately annotated, incorrectly encoded, lacks a complete set of initial conditions or parameters, or does not correspond to the accompanying model description, all of which prevents any further modifications, expansions, or reductions by a different research group.
5. *The model behavior does not correspond to what is presented in the paper*. The simulations or analyses that are reported in the article cannot be reproduced with the governing equations and/or the simulation code provided. Model repositories such as BioModels^4^ are trying to address this issue through a systematic curation process, for example, checking whether it is compliant with the Minimum Information Requested in the Annotation of Biochemical Models (MIRIAM) guidelines.^22^ This curation focuses on the correspondence of an encoded model to its associated reference description. However, it is not exhaustive for each paper, and it is limited to only a fraction of the public model space, largely because of the lack of the adequate documentation in the papers.
6. *The impact of the model is overstated*. In those cases where the modeling results can be reproduced, often the outcome does not inform the initial question(s) posed or has not been contrasted with experimental results or the authors extrapolate it to cases that are out of the model scope. Frequently the model appears to be a *post hoc* analysis of a data set rather than a novel analysis driven by a scientific question. This sheds significant doubt on whether the model is a good starting point for further decision making or model development. This is probably one of the most important challenges within the regulatory context.

The overall result from this state of affairs is a general lack of confidence in the models which coupled with their perceived highly technical nature, can undermine the trust that the bench‐based community and decision makers may have in QSP as a discipline. This situation also implies the need for a significant use of resources (industrial and academic, time‐and cost‐wise) in trying to implement, reapply, or further develop models of questionable reproducibility and/or reusability. This in turn has a negative impact on the innovation trigger phase of the Gartner Hype Cycle for QSP.

The aim of this article is to present and discuss a minimum set of recommendations that can enhance the quality, reproducibility, and further applicability of QSP models. This document summarizes the outcomes of a discussion held among academic and industrial members of the UK Quantitative and Systems Pharmacology Network (UK QSP Network).^23^ This network was created in 2015 by a group of UK and international scientists in industry and academia to bring QSP to the forefront of UK research space and nurture its growth. The network is jointly funded by the Engineering and Physical Sciences Research Council (EPSRC)^24^ and the Medical Research Council (MRC)^25^ in the United Kingdom.

These recommendations focus on the issue of how to document QSP models when published, framing a checklist of minimal requirements. Discussions about models’ accessibility, exchange mechanisms, archiving, software, and visualization or coding language are out of scope for this article.

A number of publications have recently proposed guidelines or recommendations on modeling practices. Some of these focus on the design process of QSP models,^26^, ^27^, ^28^ whereas others suggest methods for assessing the quality and/or adequacy of QSP models in particular^27^, ^28^ or models that support the MID3 paradigm in general.^29^ If physiologically‐based pharmacokinetic (PBPK) models are considered a special case of QSP modeling, then it is important to also mention the recent efforts by both the European Medicines Agency (EMA)^30^, ^31^ and the FDA.^22^, ^32^

## PURPOSE AND CONTEXT OF THE MODEL

When developing a computational model, the following two overriding questions need to be asked: “Do I need a model?” and “What is the purpose of the model?” It is tempting to become too involved in the details of the structure and implementation of a model early on in the modeling process. However, careful thought needs to be given to the aims of developing the model and, crucially, to the biological or biomedical questions that it should answer. Stepping back and asking these questions is important because they require the modeler to think carefully about the operational approach that will be taken, what data might be needed, which assumptions might have to be considered and critically, and what hypotheses will be tested. Models offer the possibility to explore relatively quickly scalable “what if” questions, serving as an extra component in the toolbox of the bench scientist to optimize their experimental procedures, thus saving time and resources on experiments that may prove unproductive. A good example of this is the use of models to reduce, refine, or replace (the “3Rs”)^33^ certain types of preclinical experiments. At the point of publication, the specific question that the model was developed to answer and/or the hypothesis that it was built to test should be clearly stated (e.g., “We tested the hypothesis that negative regulation of Syk protein phosphorylation by Tula2 leads to periodic (oscillatory) time dependent solutions”). Clarifying this information will enable potential end users of the model to determine whether it is suitable or not for their own objectives, and in the latter case, how much effort it would take to modify it and make it fit for purpose.

To address this component of the modeling process, a critical aspect to success is the inclusion of stakeholders in the development of the model. Gadkar *et al*.^28^ already refer to this important step in the development of systems pharmacology models. There are usually two types of stakeholders in the process who provide different types of input.

The first type of stakeholder is the “end user.” This person will be making use of the model simulations or analyses to gain insights into the system under study, optimize the experimental protocol, inform his or her own work, or drive decision making (including regulatory submissions). The end user is able to provide the appropriate context for the model and, as with any project that involves delivery of a “product” to an intended user, he or she should be consulted at various points during the development of the model. By defining the context, this stakeholder allows for the precise distillation of the questions to be asked of the model. In addition, an idea of the impact that the modeling exercise will have should be provided in terms of the strategic go/no‐go decision making, time reduction and/or economical costs, or societal benefit. The following is a good question to ask to elicit this information from the stakeholder: “What will happen if we don't build this model?”

The second type of stakeholder is the “domain expert.” This person can assist the modeler in understanding the mechanistic aspects of the system that is being modeled. Often what is modeled is complex and requires specific knowledge to help understand what is known and, critically, what is unknown about the system. This interaction helps to identify the assumptions that are needed or new data that may need to be found to create a model that is an appropriate abstraction and representation of the system under study. During this time, it is essential that a shared understanding of the terms used in the process is agreed on, as often the same word can be interpreted differently by different people. Sometimes the “end user” and the “domain expert” are one and the same person. Because domain expert knowledge can come from a variety of sources and elicitation approaches, consideration should be given to how this prior knowledge has been handled and combined (e.g., through a Bayesian approach as presented by Weber and Koch^34^, ^35^ in an EMA public workshop on the extrapolation of efficacy and safety in medicine development).

As proposed by Timmis *et al*.,^26^ QSP modelers could elicit and document the stakeholders’ input by borrowing a methodology from the area of safety engineering to develop robust argumentation structures. In his presentation of a model qualification method (MQM^©^) for mechanistic physiological models, Friedrich^29^ presents a series of questions that could potentially apply to more general QSP model development and lead the conversations between modelers and their stakeholders. These could be complemented by the set of example questions summarized in Table 1 in the Peterson and Riggs publication^11^ about the impact of QSP on the regulatory domain.

**Table 1:** Mock example of good practice in parameter value reporting in tabular form

Name	Definition	Value	Units	Source	Details
k	Second‐order rate constant of degradation of the inactive form upon interaction with the active form	1	(μM hour)^−1^	Smith *et al*. (2002)	The rate constant governing the interaction between the active and inactive proteins is reported to be in the order of 278 M^−1^ s^−1^ for this class of proteins
α	First‐order rate constant of inactive protein decay	0.2	hour^−1^	Doe *et al*. (2017)	Table 3 shows the half‐life values measured for inactive proteins. We took the geometric mean of those values and derived the rate constant with the expression α = ln(2)/half‐life
β	Zero‐order synthesis rate of the inactive protein	0.5	μM/hour	Derived	At steady state, β = a[α–kb] = 5[0.2–1.0.1] = 0.5
γ	Zero‐order synthesis rate of active protein	0.005	μM/hour	Derived	At steady state, γ = δb = 0.05·0.1 = 0.005
δ	First‐order rate constant of active protein decay	0.05	hour^−1^	Boggs *et al*. (1990)	Calculated from Figure 3 in the reference
*A*_0_	Initial concentration of inactive protein	5	μM	Grundy (2004)	Assumed equivalent to the average concentration of unphosphorylated Syk in untreated cells
*B*_0_	Initial concentration of active protein	0.1	μM	Plakket *et al*. (2000)	Approximated from the average total of phosphorylated and unphosphorylated ERK, assuming the active protein correlates with that of phosphorylated ERK

ERK: extracellular signal‐regulated kinase; Syk: spleen tyrosine kinase.

The justification of the type of modeling approach selected and the way it has been implemented should be derived from the questions and context provided by the stakeholders. Modelers can easily tend to favor those methodologies with which they are most experienced and comfortable instead of evaluating which approach is most adequate, such as algorithmic or mathematical, based on ordinary or on partial differential equations, deterministic or stochastic, or a hybrid between different methods. If this choice is not properly considered, then the model may not provide useful and/or correct answers. In their article on QSP design and estimation methodologies, Ribba *et al*.^27^ describe a good example of how the need for a QSP model arises from a question that cannot be solved by standard modeling approaches, such as empirical pharmacokinetic/pharmacodynamic (PKPD) modeling.

All of the information gathered during these initial phases of the modeling process should be communicated in a transparent way in the resulting publication, from a clear formulation of the questions addressed to their context, expected impact, and the reasons behind selecting a particular technical approach.

## MODEL STRUCTURE AND MODELING METHODOLOGY

After defining the purpose and context of the model comes the model‐building phase. A few initial decisions are made regarding the model structure and methodology, which do not require the use of experimental data. This section proposes good practice guidelines when reporting these decisions, which include the choice of model domain, structure, scale, modeling method, and software. The term “modeling” refers to both mathematical and computational modeling unless otherwise stated. The term “development” infers the symbolic formulation of a mathematical model or the algorithmic articulation of a computational model.

**Figure 2:**
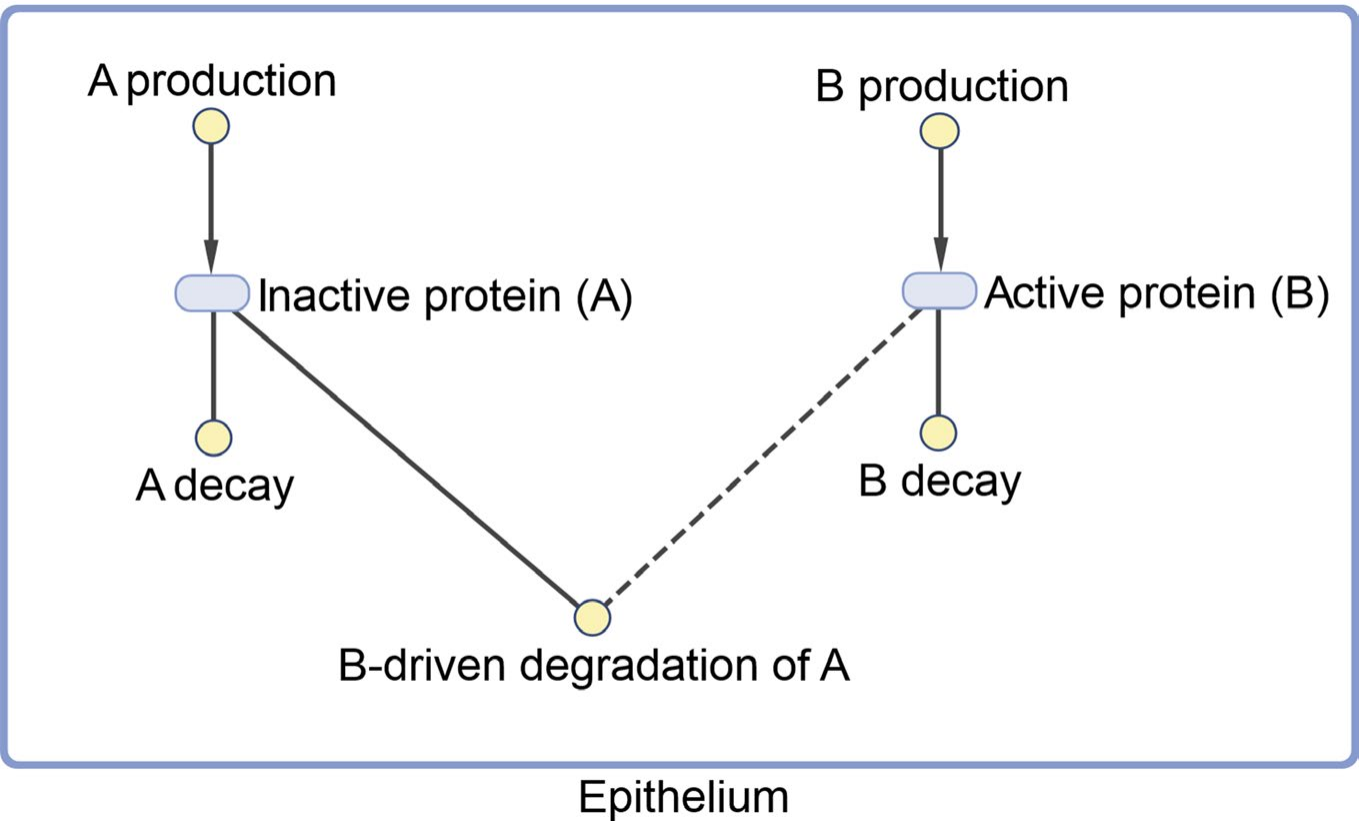
Example of good practice in model structure visualization.

1. *The model domain and general structure*. The domain to which a model is to be applied should be clearly stated in the introduction to any work, as it influences other aspects of the modeling process such as model formulation and assumptions.^28^ “Domain” may have different contextual meanings. For example, it may represent (i) a therapeutic area (e.g., neurodegeneration, oncology, etc.), (ii) biological scales (organ/tissue level, cellular level, intracellular level, or multiscale), or (iii) a specific system to which the model applies (e.g., mammalian cells, a severe asthma patient, etc.). The model purpose, as defined in the previous section, plays a significant role in determining the model domain and in explaining it to other model developers and users. In addition, the biological knowledge elicited from stakeholders determines how all of the different components of the model are connected. The domain and general structure of the model should be made clear to the reader by using some form of schematic representation (see an example in **Figure** 2), showing the most significant elements of the model, such as compartments, species, processes, feedback loops, modules, submodules, and so on. Currently, several open source and commercial software packages provide tools to produce visualizations of the model structure. Whenever possible and appropriate, it is recommended to follow some sort of standard graphical notation, such as the systems biology graphical notation (SBGN), to facilitate the reader's comprehension.^36^
2. *Model formulation or algorithm*. These recommendations depend on whether the method selected is mathematical (e.g., system of ordinary differential equations) or computational (e.g., agent‐based models). For mathematical models, the formulation of the model needs to be correctly stated using relevant notation and explanations. All dependent and independent variables need to be accompanied by written descriptions (e.g., “Here x(t) represents the total concentration of the metabolite, and t is time”), with explicit statements of dependencies, for example, on time. All equations need to be clearly displayed on the page with relevant boundary and/or initial conditions stated. The mathematical domain of problem definition also ought to be stated (e.g., “∀t > 0”). A written explanation of each term in the equation should be given either before or after the stated mathematical formulation and/or overarching explanations of the biological or pharmacological meaning of each term stated (see **Box** 1 for an example). This should include any abstractions and/or simplifications made. In the case of a computational model, all of the above applies to any equations associated to the algorithm, and the latter needs to be clearly stated using pseudo‐code. For instance, in the case of an agent‐based or cellular automata model, the definitions of all parameters and rules describing how each individual element of the model behaves need to be indicated. The transition between each state at each given temporal point or spatial point needs to be made clear (e.g., “individuals move between proliferative and apoptotic states at probabilistic rates selected randomly from a uniform distribution”), including the condition that triggers each transition (e.g., “where the selected rate is less than 0.5”).^26^ In both mathematical and computational models, all definitions need to include a correct statement of the parameter units (generally from the International System of Units), their value (or range of values, if variability is considered), and source (for further details, see the “Input Data, Knowledge, and Assumptions Going Into the Model” section). Where space and word limitations do not allow for this information to be in the article main text, it can be provided as supplementary materials.
3. *Model solving and simulation method*. For mathematical models consisting of differential equations, these need to include a statement on the algorithm used to solve the governing equations and the value of algorithm‐relevant parameters for doing so (e.g., “The governing ordinary differential equations were solved using the Runge‐Kutta fourth/fifth order method implemented via the ode45 solver in MATLAB^®^. The relative tolerance was set at 1 × 10^−6^”). The type of machine, platform, and, where applicable, package used to solve the equations needs to be stated (e.g., “The governing equations were solved using MATLAB version 2017a on an Apple iMac desktop running macOS Sierra Version 10.12.6”). The steps used by a computational model algorithm need to be made clear. Where models are considerably complex, pseudo‐code outlining the main steps should be generated, ideally via a schematic diagram or plan.
4. *Code files*. The recommendation is to make available to other users the code and/or model files generated to build and solve the models. This can be done in several ways, ideally by depositing them in online model repositories such as BioModels,^4^ CellML,^5^ or DDMore,^6^ which provide a platform for easy model search and retrieval. Other ways to share models include the provision as supplementary material of scientific articles, the uploading of copies to academic author websites, and the sharing on public platforms for computational code such as GitHub.^37^ The best approach is to encode a model in standard formats when possible (e.g., SBML,^9^ CellML,^38^ PharmML,^17^ or MDL^18^ in the case of ordinary differential equation systems), as they allow interoperability.^16^ The code should be easy to follow, annotated and as error free as possible. Ulterior users should check it for consistency before using it, and it would be in the best interest of the modeling community to report any errors found to a designated member of the research team (e.g., corresponding author) or to the curators of the model repository. Future maintenance and the upgrading of the code by the original authors should not be a stipulation of it being deposited in repositories.

Box 1 **Example of good practice in mathematical formulation**

The interaction between the inactive protein *A*(*t*) and its active form *B*(*t*) is given by the following governing equations:

$\frac{\text{dA}}{dt}=\overset{\text{Inactive protein production}}{\overbrace{\beta }}-\overset{\text{Inactive protein degradation via active form}}{\overbrace{\text{kAB}}}-\overset{\text{Inactive protein decay}}{\overbrace{\alpha A}},$

$\frac{\text{dB}}{dt}=\overset{\text{Active protein production}}{\overbrace{\gamma }}-\overset{\text{Active protein decay}}{\overbrace{\delta B}}\forall t>0,$

with the initial conditions *A*(0) = *A*_0_ μM and *B*(0) = *B* μM, where *t* represents time in hours. Here *k* is the reaction rate constant in (hour μM)^−1^ units representing the rate at which the degradation of 1 μM of inactive protein (A) is triggered by its interaction with 1 μM of the active protein (B), α is the rate constant of inactive protein decay (hour^−1^), β is the production rate of the inactive protein (μM/hour), γ is that of the active protein (μM/hour), and δ is the rate constant of active protein decay (hour^−1^).

## INPUT DATA, KNOWLEDGE, AND ASSUMPTIONS GOING INTO THE MODEL

Once the model structure and methodology have been defined and justified, the model then needs to be connected with experimental observations for it to fulfil its purpose. This means, first, informing the variables and parameters in the model with specific values in a process that is generally known as “model parameterization”. Second, experimental data are required to gain an idea of the quality of the performance of the model by comparing it to the known behavior of the biological system. This section deals specifically with the former aspect of experimental data usage.

The reproducibility of modeling results depends on the specific parameter values used, thus the reporting of those values, how they were determined, and the source of information for doing so is of high importance. A parameter description (most often in tabular form; see an example in **Table** 1) should be supplied, including the symbol or name representing the parameter, its definition, value, units, and the sources used to determine it. Details of how the parameter value was obtained need to be reported, specifically where parameter values have been derived as functions of other parameters not included in the model or by fitting model simulations to experimental data. Such parameter estimations usually yield statistical information related to parameter precision (importantly, not accuracy) such as standard errors or correlation matrices, and sometimes information derived from more sophisticated approaches such as profile likelihood.^39^ All of this information could be added to the parameter table and/or in an annex (e.g., “Parameter values were first determined by assuming equal rates of absorption and excretion. These were then varied to obtain a least‐squares fit between the model and the patient cytokine levels reported by Smith *et al*. The parameter values reported are those that gave the minimal error residuals”). It is important to note that the concept of “parameter” here is used in its widest meaning and includes scalars such as compartmental volumes as well as amounts or concentrations of the interacting species in the system at steady state or at initial conditions.

Input data can come from a variety of sources. It can be extracted from public or in‐house literature or databases, obtained from a personal communication (which ideally should be referenced), or from experiments specifically designed to inform one (or a subset) of the processes represented in the model. Data can also come in a variety of types, including kinetic or thermodynamic constants measured *in vitro* or *ex vivo*, physiological parameters, biomarker clinical data at pretreatment or from longitudinal studies (including ‐omics data), drug pharmacokinetic profiles or parameters (fitted or allometrically scaled from *in vivo* parameters), and drug safety end points. Whatever the source and form of the data, details of the key questions that drove the experiment and the experimental conditions should be explicit and relevant to the system being modeled. Limitations of the data, such as known errors, outliers, high variability, or decisions on the removal of particular data points or data sets, should also be stated. Input data can be provided along with the code file as supplementary information or as a reference to a repository for unstructured data such as BioStudies.^40^

Knowledge in the context of QSP model parameterization refers to the qualitative and/or semiquantitative information that the scientific community in general and the modeler stakeholders hold about the expected behavior of the model or the biological processes described therein. For example, a certain marker of drug activity may be known to reach a peak after about 3 hours of treatment and decline to baseline levels after 12 hours, but no explicit data set is available to fit the model parameters describing that activity.

Assumptions come into play when there is not enough data available to parameterize the model and existing knowledge about the system is insufficient to inform parameterization or to reduce the number of parameters. The setting, testing, and evaluation of model assumptions have been previously referred to and described in the context of the MID3 paradigm.^41^ They have been divided into the following six distinct types of assumptions: pharmacological, physiological, disease, data, mathematical, and statistical. Assumptions are scoped by what the model is intended for (e.g., to understand process relationships, to simulate the effect of different scenarios on pharmacological events), as discussed in the previous section. Care should be taken to keep the number of assumptions as low as possible because an increase in the number and complexity of modeling assumptions can lead to higher uncertainty and risk around modeling results. The level of evaluation and testing of the assumptions (e.g., by sensitivity analysis, discussed in the next section) needs to be tailored accordingly. This is a key aspect in the selection of the right level of granularity in a QSP model which, as Ribba *et al*.^27^ point out, is one of the most difficult decisions during the QSP modeling process.

Careful documentation of the collation of sources and usage of input data, knowledge, and assumptions made in formulating and informing a model increases the confidence in its results. It acts to ensure that quality assurance and quality control of the developed model have been undertaken.^41^, ^42^ A discussion of the potential limitations of the model in the context of the available data, knowledge, and assumptions also constitutes good practice because it allows modeling practitioners to reuse or extend the QSP model with new or expanded information.

## MODEL VERIFICATION

The definition of model verification in the literature is rather unresolved. Some authors describe model verification as the part of a model qualification process that focuses on the correctness of the mathematical model structure.^31^ They understand model qualification as a broad activity aimed at establishing confidence in the model to simulate a certain scenario in a specific context and showing its ability to predict with a certain purpose. Model qualification has also been described as an approach to determine whether a model is fit for purpose.^29^ On the other hand, other authors^30^, ^32^, ^43^, ^44^ define verification as the process of model testing with a data set that has not been used for initial model building, often part of a verification/modification iterative process of model refinement prior to its application. Here, we refer to model verification as the process in which the modeler determines the degree of self‐consistency and robustness of the model prior to assessing its performance against experimental observations.^43^, ^45^

At the very least, computational and mathematical models solved numerically should have their code tested for consistency. Such testing consists of ensuring the software code does not contain any coding errors (e.g., *bona fide* mistakes or additionally coded terms not included in the original formulation/statement) and checking that the solutions to known results or limit conditions of the model (e.g., A  +  B → C yield no C when A or B are zero) are correctly produced. Such testing can greatly alleviate the likelihood of undertaking experimental work based on model predictions that are later found to be incorrect.

In addition, mathematical and statistical analyses can help us understand the range of applicability of a QSP model; it provides tools to allow us to explore behavior for which there is a lack of data. In fact, the analysis of any model is important in identifying a system's overall behavior. Such an analysis can highlight aspects not previously conceived or considered and help provide a comprehensive understanding of the system dynamics. The reporting of the mathematical analyses of a model should include the methods of analysis used and outline the main steps required to reproduce the results. These results should be clearly stated and their meaning placed in the context of the overall problem/application considered.

There are certain key tests and analyses for QSP models that should be considered when performing model verification. One of them is structural identifiability analysis, which can help determine whether the model structure and parameter estimates inferred from the known or assumed properties of a system are suitable. This can help determine whether increasing the quality of the input data set used for parameter estimation (see the previous section) would help reduce uncertainty in the estimation, the uncertainty may stem from a poor model structure. This analysis considers the uniqueness (or otherwise) of the unknown model parameters from the input‐output structure corresponding to experiments proposed to collect data for parameter estimation. Such an *a priori* analysis applies under an assumption of the availability of perfect, noise‐free observations. This is an important but often overlooked theoretical prerequisite to experiment design, system identification, and parameter estimation because estimates for unidentifiable parameters are effectively meaningless. If parameter estimates are to be used to inform about intervention or inhibition strategies or other critical decisions, then it is essential that the parameters be uniquely identifiable or otherwise supported from independent, reliable information. This is particularly true if the model potentially may incorporate many unknown model parameters with relatively few model outputs. Numerous techniques for performing a structural identifiability analysis on linear parametric models exist, and this is a well‐understood topic.^46^, ^47^, ^48^ In comparison, there are relatively few techniques available for nonlinear systems, and significant (symbolic) computational problems can arise, even for relatively simple models.^49^, ^50^, ^51^ During the past few years, software packages have been developed and are generally widely available that can ease the burden in performing such analysis.^52^, ^53^, ^54^, ^55^ These include (and are not limited to) structural identifiability analysis software using the exact arithmetic rank approach (EAR),^56^ StrikeGoldd2,^57^ COMBOS,^58^ DAISY^59^ and Profile Likelihood.^39^ In addition, there are also techniques available in the literature^60^, ^61^ to establish model parameter redundancy, which alongside structural identifiability analysis can support model reparameterization or lumping, should this be required.^62^ The introduction of recent techniques for the structural identifiability analysis of mixed‐effects models (commonly applied in a population PKPD context) widens the scope of the application of such analysis to QSP models.^54^ With the availability of such tools, there should be an expectation that a certain level of structural identifiability analysis of QSP models should be performed and reported as a prerequisite to parameter estimation and as a component of experiment design.

In the case of dynamical mathematical models (e.g., differential equations), one should also seek to determine the steady states of the system. In a QSP model, these should include the steady states in absence of drug. This allows two things to be achieved. First, such analysis acts as a consistency check—is the model reproducing the baseline state? If not, is the model then correctly formulated? Second, it allows for a basic understanding of the system to be obtained. If multiple steady states are found, then changes in the parameters can lead to changes in the stability or even existence of these steady states, which can lead to dramatic changes in the overall dynamics of the model. A bifurcation analysis determines those curves in the parameter space at which such changes occur. If certain dynamics are not biologically feasible, then this gives bounds on the feasible parameter regions and thus helps determine when an extrapolation is likely to work and when it is probably going to fail. In addition, such an analysis can also aid parameter estimation. If such transitions have been observed experimentally, this can provide an implicit method to determine unknown parameters. For small‐scale models (e.g., four governing differential equations or fewer), such an analysis can generally be achieved analytically. For larger scale systems, such work can be undertaken using mathematical computer packages such as Maple,^63^ MATLAB^®^,^64^ and DsTool.^65^ Model reduction methods should also be considered as a means of gaining further understanding of the system.^66^

Computational analysis of mathematical models should also be undertaken as a complement to the above analysis techniques or where such mathematical analysis is not possible. At the very least, the analysis of dynamic models (mathematical or computational) should include a sensitivity analysis. Sensitivity analysis is commonly applied to parameterized models to establish those parameters that are most sensitive to perturbation and those that have the most effect on model responses when perturbed. Formal sensitivity has long been considered an important aspect of PKPD modeling (see, for example, Nestorov *et al*.^67^, ^68^). Kirouac^69^ even suggests a methodology, underpinned by sensitivity analysis approaches, that could become a means to quantifying qualitative QSP simulation results, placing this technique in the realm of model “validation.” Clearly such an analysis is also a key component in the modeling of QSP systems, and with techniques such as generalized sensitivity functions, should be a fundamental component in QSP modeling.^70^ Indeed, a growing number of journals require such analysis be undertaken before a manuscript is accepted for publication.

The robustness of model simulations to input perturbations should also be considered, potentially including μ‐analysis, an approach to quantifying the effects of parameter uncertainty as applied to models in systems biology, for example, to ascertain the extent of applicability of the models generated.^71^ This may also be performed in a statistical context.

However, there will always be uncertainties in the parameter estimates even if the model is structurally identifiable and the quality and quantity of the experimental data are relatively high. The combined effects of dynamical model analysis (analytical and computational) and identifiability analysis can be greatly informative in assisting the design of further experiments to inform on unidentifiable parameters and their values without the need for a reduction of the model and thus loss of descriptive power. As well as this, an approach to further strengthen the plausibility of the model simulations is to use experimental data to validate the model.

## MODEL VALIDATION

We refer to model validation as the process in which the modeler determines the degree to which the model is an accurate representation of the real world^43^, ^45^ using experimental observations that have not been used as input to estimate the model parameters.

In the context of QSP, this is equivalent to running a multiscale simulation of the model, where emerging properties of the whole or of a submodule of the model are compared with the behavior observed experimentally for that model or submodule. The validation of individual model submodules (e.g., the cell cycle of eosinophils in the human body, or the absorption, distribution, metabolism and excretion (ADME) cycle that a drug undergoes in a healthy volunteer) adds confidence in the parameter values for those submodules, whereas the validation of the whole model high‐level behavior (e.g., the forced expiratory volume in 1 second (FEV1) response in asthmatic patients under a specific drug treatment) adds confidence on how those submodels were integrated with each other and within the framework of human physiology. The latter is also the ultimate measure of credibility of the simulations that will address the specific questions posed initially by the modeler.

As mentioned in the “Input Data, Knowledge, and Assumptions Going Into the Model” section, experimental observations can consist in data or in knowledge about the behavior of the biological system. Where quantitative data are available to assess the degree of performance of the model, this should be utilized. When no quantitative data are available, qualitative data and/or “expert” stakeholder knowledge of the system trends can be applied, for example, to rule out certain hypotheses about the intrinsic behavior of the system. In either case, at the point of publication, the data or knowledge used to validate the model should be clearly referenced and described and its relevance to the model context adequately explained. Lu *et al*.,^72^ Kanodia *et al*.,^73^ Ortega *et al*.,^74^ Karelina *et al.,* 75 and Peterson and Riggs^76^, to name some examples, illustrate well the validation of a model and its reporting.

The modeler should aspire to produce plots of simulations and overlay the experimental data onto these, including any measures of their potential and perceived variability (i.e., standard error bars or 95% confidence intervals around the experimental data, shadows from ensemble simulations, etc.) whenever possible. As more data sets and increasingly diverse contexts and types of perturbations are compared with the model simulations, the more opportunities arise to learn about the domain of applicability of the model. The term “learn” here is substantially important—models are not only useful for their potential to predict the system behavior but also essentially for consolidating the knowledge and assumptions that exist about the system. A disagreement between simulations and experimental observations, assuming experimental observations can be considered to be directly pertinent, highlights the fact that one or some of the underlying assumptions in the model are incorrect.^77^ Thus, the model structure and/or its parameter values need to be reconsidered and potentially new experimental assays designed to fully understand from where the disagreement stems. Often, the trends in residual analysis, or qualitative features of the simulation, can help spot those assumptions that are potentially incorrect. Once those disagreements have been minimized or when the specific situations where those disagreements appear have been clearly identified, then confidence in the model simulations, the underlying assumptions, and the overall credibility of the model will increase.

Model quality tests usually run in the context of other *in silico* simulations, such as Akaike's information criterion, Bayesian information criterion, *R* ^2^, *Q* ^2^, and so on may be inadequate here because the goal of QSP models is not to obtain a highly precise estimate of the system readouts (e.g., biomarker levels changes), but to obtain a ballpark estimate with the limited knowledge that there is of the system components, of what can be expected when all of those components are put together.^78^

Ultimately, the validation of the model ought to be performed in the strictest sense of the term validation: this is when the data available to test the model performance have been generated in an experiment designed on the basis of the model simulations. However, it is unusual at the time of publishing the model for the modelers to count on these data, so normally the reporting of the modeling exercise finishes with the application of the validated model to resolve those questions posed by the “end user” stakeholders.

## MODEL RESULTS, APPLICATION, AND IMPACT

Once the model has been developed and has reached a satisfactory degree of maturity and performance, it can then be applied to answer the questions originally posed. The degree to which the model can answer those questions and the answers themselves will determine its impact.

The answers normally rely on some sort of graphical representation of the model simulations and on the capture of the outcome values of key system elements (e.g., regarding molecular species, cell types, system agents, clinical biomarkers, etc.) during and at the end of those simulations. All of these data, graphical or numerical, should be reported clearly as well as the model conditions in which those simulations were run, including initial conditions and simulation time.

The impact of the model is primarily measured in relation to the original purpose of the modeling exercise, that is, the questions posed and the anticipated output. The impact, including its type and extent, will be viewed differently in different types of environments (academic, industry, or regulatory) and by different types of roles (bioscientist, medic, pharmacologist, chemist, practitioner, manager, director). It is for this reason that regulatory vs. industry criteria for impact have been developed.^41^, ^79^

With respect to direct impact within our original list of stakeholders, for the “end user” the impact will be judged by whether key questions have been addressed and whether the model can be easily interrogated to answer emerging questions. For the “domain expert”, judgment may well be based on whether the model “sensibly” encodes biological knowledge. The assessment of the model's impact by the modelers will be partly based on feedback from the stakeholders, but direct impact will depend on whether the modeling exercise has provided a reusable model and a deeper understanding of how to model a particular aspect of biology and pharmacology. It is important to note that the perception of added value that the “end user” stakeholder has of the modeling exercise is instrumental in achieving and measuring impact beyond the initial circle of influence.

Regarding the knowledge acquired, the value added by the QSP model could be the following:

1. A positive new discovery: the model allows the exploration of untested contexts, so it could become a source of new (ranges of) parameter values (e.g., “what is the required potency for the new drug?”) or of a completely new hypothesis (e.g., “what if we test this new dosing regimen?” as was the case with the rhPTH filing^11^).
2. A confirmation: the model may not venture into extrapolating to untested contexts, but because of its integrative, bottom‐up nature, QSP provides unique insights that are unavailable through alternative modeling techniques, so it becomes a sandpit where existing data can be interpreted in data‐rich contexts or where uncertainty can be explored and delimited in data‐poor contexts, enabling better informed decisions.
3. A realization of a misconception: when a modification of model assumptions is required to describe experimental data, this points to a gap in biological knowledge (e.g., “our current understanding of the drug target biology does not explain the observed *in vitro* phenomena quantitatively”), which generates a revision of the initial hypothesis; the QSP model can then help identify the key questions needed to expand our knowledge sufficiently and design the studies required to inform them.^77^

The type of decision making that is affected by this knowledge will depend on the specific environment where the decision is made. In a pharmaceutical business, the knowledge acquired could translate into, for example, stopping or starting a project, seeking the reoptimization of certain candidate drug properties, or redesigning an experiment or clinical study. A good example of the latter was reported by Entelos^80^ in their publication^78^ about the optimization of a phase I clinical trial protocol for a first‐in‐class therapy against type 2 diabetes mellitus (T2DM): their QSP simulations led to the reduction from a “6 arms for 14 weeks” to a “2 arms for 8 weeks” design. In academia, the newly acquired knowledge could perhaps lead to the decision of developing a new research program, seeking a collaboration with research teams of bench scientists, or publishing a high‐impact, peer‐reviewed article. In a regulatory setting, the knowledge derived from a QSP model can translate into an approval (e.g., the rhPTH example^11^) or a rejection of the license application.

The impact of a QSP modeling exercise can have a ripple, multifold effect beyond the modeler's initial stakeholders. The success of modeling is especially compelling when impact can be translated into financial figures, reflecting any sort of cost or time savings in a business, in an academic institution, or even in the national budget. The article on the T2DM QSP model mentioned previously^80^ is an example of reporting this sort of impact: the authors estimated a reduction in 66% of the cost and 40% of the time length of the phase I trial. In the context of modeling, albeit not exclusively of QSP, there are a couple of significant examples by Pfizer^81^ and Merck.^82^ Finally, there is the societal impact, including the enhanced 3Rs of animal usage,^83^ reduced patient burden, or development of scientific talent among others.

Several groups have collated the impact of modeling and simulation, although only a limited subset of these would be considered QSP. The impact examples collected by the American Society for Clinical Pharmacology and Therapeutics (ASCPT) Quantitative Pharmacology Network^84^ provide a number of case studies, including the key question that needed to be addressed, impact on decision making, and estimated time saving. Of note are the translational medicine examples that generate predictions before clinical data are generated, including the optimization of bispecific antibody affinities and the translation of the efficacy of antimicrobial agents using nonclinical data.

Reporting the impact of each QSP model developed and published, at all of these different levels, is of crucial importance if the QSP community aims to widen the adoption of this discipline.

## CONCLUSIONS AND FUTURE WORK

This article attempts to summarize the considerations made by members of the UK QSP Network on good practice for the conduct and reporting of each stage of QSP model development and application. QSP is a very useful body of technologies but also a highly technical area, so the communication of both methods and results is of the utmost importance. In particular, sufficient information regarding the following should be given to enable the work to be reproduced:

1. The questions and objectives that the modeling exercise set out to answer and achieve
2. The modeling assumptions (biological and mathematical)
3. The sources of data and prior parameter values
4. The mathematical model structure
5. Final parameter values
6. The existence of steady states and/or oscillations
7. The computational algorithms used
8. The conclusions that the modeling supported

A summary list of all recommendations, along with references, can be found in **Table** 2.

**Table 2:** Abbreviated list of recommendations on best practice to maximize the use and reuse of QSP models

QSP workflow step		Recommendations		Relevant references
		Mathematical	Computational	
1. Purpose and context of the model		Ask “Do I need a model?” and “What is the purpose of the model?” Engage with stakeholders: “end users” and “domain experts” Formulate clearly the questions addressed, their context, expected impact of the decisions derived from the model, and rationale for the selection of QSP as modeling methodology		Peterson & Riggs (2015)^11^ Timmis *et al*. (2017)^26^ Ribba *et al*. (2017)^27^ Gadkar *et al*. (2016)^28^ Friedrich (2016)^29^
2. Model structure and modeling methodology	i. Model domain and general structure	Define clearly the model domain: therapeutic area, biological scale, biological/clinical system Provide a schematic representation of the model domain and general structure (e.g., **Figure** 2) Whenever possible, follow standard graphical notation (e.g., SBGN)		Gadkar *et al*. (2016)^28^ **Figure ** 2 Le Novère *et al*. (2009)^36^
	ii. Model formulation or algorithm	Provide all equations and boundary conditions (e.g., **Box** 1) Explain all the terms and their biological/pharmacological meaning	Clearly state the algorithm using pseudo‐code and clearly state any associated equations Explain all the rules and parameters and their biological/pharmacological meaning	**Box ** 1 Timmis *et al*. (2017)^26^
		Explain any abstractions and/or simplifications made Report units for each element in the model		
	iii. Model solving and simulation method	State the method used to solve the system of equations (e.g., Runge‐Kutta fourth/fifth order implemented via the ode45 solver in MATLAB^64^) Provide absolute/relative tolerance value	Clearly state simulation engine used (and version)	Timmis *et al*. (2017)^26^
		Provide software package used and version		
	iv. Code files	Share code and model files generated to build and run the model via the following: ○ Supplementary material of an article ○ Public online model repositories (e.g., BioModels^4^, DDMore^6^) ○ Academic author websites, or ○ Public platforms for computational code (e.g., GitHub^37^) Ensure code is easy to follow, adequately annotated, and as error free as possible Whenever possible, use a standard format (e.g., SBML, PharmML)		Chelliah *et al*. (2015)^4^ Lloyd *et al*. (2008)^5^ DDMore‐Foundation (2012–2018)^6^ GitHub (2018)^37^ Hucka *et al*. (2003)^9^ Golebiewski (2019)^16^ Swat *et al*. (2015)^17^ Smith *et al*. (2017)^18^ Cuellar *et al*. (2003)^38^
3. Input data, knowledge and assumptions going into the model		Use input data from systems under experimental conditions as relevant as possible to the system being modeled Provide a detailed model parameter description, including the following: ○ Symbol/name of parameter ○ Definition ○ Parameter value (or range of values) ○ Units ○ Sources used to obtain it (literature citation, database, derivation from other parameters, experiment presented in the same report/article, *in silico* estimations, etc.) ○ Details of how the parameter value was determined (measured directly, fitted or assumed) and whether the underlying data has any limitations (suspected errors, outliers, high variability, excluded data points, etc.) Consider using a tabular format to present this information (e.g., **Table** 1) Consider providing actual data files along with code files (see 2. Model structure and modeling methodology, iv. Code files in this table) Describe the following in detail: ○ Qualitative and/or semiquantitative knowledge obtained firsthand from stakeholders ○ Assumptions (pharmacological, physiological, disease, data, mathematical, statistical) and how they were tested Discuss potential limitations of model in the context of available input data, knowledge, and assumptions		**Table ** 1 Sarkans *et al*. (2018)^40^ Marshall *et al*. (2016)^41^ Ribba *et al*. (2017)^27^ Bonate *et al*. (2012)^42^
4. Model verification		Test code for consistency: ○ Eliminate detected coding errors ○ Ensure solutions or limit conditions reached by the model are correct (e.g., A + B ‐> C yields no C when A and B are set to zero) Determine the steady states of the system Run a sensitivity analysis to identify which parameters have the most effect on model responses and how significant is that effect		Anderson *et al*. (2007)^43^ Hicks *et al*. (2015)^45^ Nestorov *et al*. (1999)^67^ Nestorov *et al*. (1997)^68^ Kirouac (2018)^69^ Thomaseth & Cobelli (1999)^70^
		When model parameters are assumed, that is, not supported by independent, reliable input data or knowledge (see 3. Input data, knowledge and assumptions going into the model in this table): ○ Check that those parameters are identifiable ○ Consider techniques to establish model parameter redundancy Consider running a bifurcation analysis to define the scope of extrapolations from the model Consider model reduction methods		Walter *et al*. (1987)^48^ Janzen *et al*. (2016)^53^ Raue *et al*. (2014)^55^ Karlsson *et al*. (2012)^56^ Villaverde *et al*. (2019)^57^ Meshkat *et al*. (2014)^58^ Saccomani *et al*. (2010)^59^ Choquet *et al*. (2012)^60^ Cole *et al*. (2010)^61^ Back *et al*. (1992)^65^ Snowden *et al*. (2017)^66^
5. Model validation		Describe and clearly reference the data or knowledge used to validate the model and explain its relevance to the model context Plot model simulations overlaying the corresponding experimental data onto them with measures of potential/perceived variability (e.g., standard error bars, confidence intervals, shadows from ensemble simulations)		Anderson *et al*. (2007)^43^ Hicks *et al*. (2015)^45^ Lu *et al*. (2014)^72^ Kanodia *et al*. (2014)^73^ Ortega *et al*. (2013)^74^ Karelina *et al*. (2012)^75^ Peterson and Riggs (2012)^76^ Agoram (2014)^78^
6. Model results, application, and impact		Articulate a clear answer to the questions originally posed for the model (see 1. Purpose and context of the model in this table) Provide the simulation plots and/or outcome numerical values that underpin those answers Qualify the type of knowledge acquired through the modeling exercise: a positive new discovery, a confirmation, and/or a realization of a misconception. Describe the decisions that the modeling exercise enabled for the different stakeholders (user, domain expert, academic, industry, regulatory)—qualitatively and, whenever possible, quantitatively Describe the impact of the QSP modeling exercise beyond the initial stakeholders, especially if the impact is societal and/or can be translated into financial figures		Marshall *et al*. (2016)^41^ Shepard (2011)^79^ Peterson & Riggs (2015)^11^Hendricks (2013)^77^ Kansal & Trimmer (2005)^80^ Milligan *et al*. (2013)^81^ Allerheiligen (2014)^82^ Bueters *et al*. (2013)^83^ Nayak *et al*. (2018)^84^

PharmML, pharmacometrics markup language; QSP, quantitative and systems pharmacology; SBGN, systems biology graphical notation; SBML, systems biology markup language.

The widespread adoption of these good practices should facilitate not only broad adoption of QSP but also the reuse of existing QSP models in new and exciting areas of pharmacology and drug discovery. These include, just as examples, the possibility to build on existing QSP models to extrapolate the results of drug treatment between diseases with common underlying mechanisms (e.g., different cancer types), and the definition and evaluation of optimal drug combinations^85^ considering the sequence and timing of a given therapy.

An area where QSP holds much promise is in pediatric and vulnerable populations.^86^ In particular, the understanding of neonatal, obstetric, and fetal pharmacology is important. Much progress has been made in terms of extrapolation from adult or adolescent data to pediatric populations (which currently is done outside of QSP, usually via pharmacometric and PBPK approaches).^87^ However, more needs to be achieved in terms of understanding the ontogeny and maturation processes (fetal development, the placenta exchange process, neonates, pediatrics^88^, ^89^). This would also support hypotheses on similarities of disease mechanisms between different stages of development. Another area where multiscale models have potential is in their use in concert with pharmacometric models to study how an observed variability in patient response can be explained by, for example, variables influencing trial design, variation in disease‐specific covariates (not exclusively pharmacokinetics), or the complex pathway modulation that can be engendered by drug combinations. This summary will hopefully serve as a helpful guidance to QSP practitioners seeking to maximize their impact as well as to other stakeholders considering the use of QSP models in their projects.

## Funding

The authors acknowledge funding support from the Engineering and Physical Sciences Research Council and Medical Research Council for the UK Quantitative Systems Pharmacology Network (EP/N005481/1). R.S.M.‐S. has been supported by the Biotechnology and Biological Sciences Research Council with a MultiMod grant BB/N019482/1 and European Molecular Biology Laboratory core funding.

## Conflict of Interest

L.C.‐S. is an employee and shareholder of GlaxoSmithKline. S.Y.A.C. is an employee of AstraZeneca. M.P. is an employee and shareholder in Union Chimique Belge. A.P. is an employee and shareholder of Hoffmann‐La Roche. J.T. is Director of SimOmics Ltd. As Editor‐in‐Chief of *CPT: Pharmacometrics & Systems Pharmacology*, P.H.v.G. was not involved in the review or decision process for this article. P.V. is an employee of MedImmune, a wholly owned subsidiary of AstraZeneca. J.W.T.Y. is an employee and shareholder of AstraZeneca. M.J.C., V.C., G.D., R.S.M.‐S. and M.J.T. have no conflicts of interest to declare.
